# Diet Quality and Liver Health in People Living with HIV in the MASH Cohort: A Multi-Omic Analysis of the Fecal Microbiome and Metabolome

**DOI:** 10.3390/metabo13020271

**Published:** 2023-02-14

**Authors:** Haley R. Martin, Sabrina Sales Martinez, Vitalii Stebliankin, Javier A. Tamargo, Adriana Campa, Giri Narasimhan, Jacqueline Hernandez, Jose A. Bastida Rodriguez, Colby Teeman, Angelique Johnson, Kenneth E. Sherman, Marianna K. Baum

**Affiliations:** 1 Robert Stempel College of Public Health and Social Work, Florida International University, 11200 SW 8th Street, AHC-5 500, Miami, FL 33199, USA; 2 Bioinformatics Research Group (BioRG), School of Computing and Information Sciences, University Park Campus, Florida International University, ECS-254, Miami, FL 33199, USA; 3 Biomolecular Sciences Institute, Florida International University, 11200 SW 8th Street, AHC4 211, Miami, FL 33199, USA; 4 Department of Internal Medicine, Division of Digestive Diseases, College of Medicine, University of Cincinnati, 3230 Eden Ave, Cincinnati, OH 45267, USA

**Keywords:** HIV, metabolome, metabolomics, microbiome, liver disease, diet, diet quality, Healthy Eating Index, nutrition

## Abstract

The gut–liver axis has been recognized as a potential pathway in which dietary factors may contribute to liver disease in people living with HIV (PLWH). The objective of this study was to explore associations between dietary quality, the fecal microbiome, the metabolome, and liver health in PLWH from the Miami Adult Studies on HIV (MASH) cohort. We performed a cross-sectional analysis of 50 PLWH from the MASH cohort and utilized the USDA Healthy Eating Index (HEI)–2015 to measure diet quality. A Fibrosis-4 Index (FIB-4) score < 1.45 was used as a strong indication that advanced liver fibrosis was not present. Stool samples and fasting blood plasma samples were collected. Bacterial composition was characterized using 16S rRNA sequencing. Metabolomics in plasma were determined using gas and liquid chromatography/mass spectrometry. Statistical analyses included biomarker identification using linear discriminant analysis effect size. Compared to participants with FIB-4 ≥ 1.45, participants with FIB-4 < 1.45 had higher intake of dairy (*p* = 0.006). Fibrosis-4 Index score was inversely correlated with seafood and plant protein HEI component score (*r* = −0.320, *p* = 0.022). The relative abundances of butyrate-producing taxa *Ruminococcaceae, Roseburia*, and *Lachnospiraceae* were higher in participants with FIB-4 < 1.45. Participants with FIB-4 < 1.45 also had higher levels of caffeine (*p* = 0.045) and related metabolites such as trigonelline (*p* = 0.008) and 1-methylurate (*p* = 0.023). Dietary components appear to be associated with the fecal microbiome and metabolome, and liver health in PLWH. Future studies should investigate whether targeting specific dietary components may reduce liver-related morbidity and mortality in PLWH.

## 1. Introduction

Current antiretroviral therapy (ART) has greatly improved the prognosis of people living with HIV (PLWH) [1]. However, as AIDS-related deaths have declined, non-AIDS-related comorbidities have become more apparent [2]. Liver disease, in particular, has emerged as a leading cause of morbidity and mortality in PLWH [2,3], facilitated by oxidative stress, viral hepatitis, systemic inflammation, substance use such as alcohol misuse and cocaine use, and gut microbial translocation [4,5,6]. The role of the gut–liver axis has increasingly been recognized as a significant pathway that may contribute to chronic liver disease in HIV when considering dietary, genetic, and environmental conditions [4,7].

Microbial translocation occurs along the gut–liver axis when microbes and metabolites cross the epithelial barrier and travel to the liver [4]. In HIV infection, cells that maintain gut barrier integrity may be depleted [8], contributing to the translocation of bacterial lipopolysaccharides (LPS) to the liver and activation of pathways known to upregulate inflammatory and fibrotic pathways [4]. Furthermore, the composition of the microbiome has been shown to be altered in PLWH [9], and in subjects at different stages of liver disease [10]. A gut microbiome metagenomic signature differentiated adults with mild/moderate liver fibrosis from those with advanced liver fibrosis [11,12]. This metagenomic signature demonstrated robust accuracy when compared to other non-invasive measures of liver fibrosis such as the Fibrosis-4 Index (FIB-4) [12]. Loomba et al. describe a decrease in the Firmicutes phylum and an increase in Proteobacteria as liver disease progresses from non-alcoholic fatty liver disease (NAFLD) to advanced liver fibrosis [11]. Taken together, this suggests that even mild forms of liver disease may be characterized by dysbiosis, described as an imbalance of potentially pathogenic and beneficial microbiota that may have a deleterious effect on the host [13].

Diet and microbial metabolites have also been investigated for their role in mediating liver disease; certain dietary intake patterns may contribute to the initiation and progression of liver diseases such as excessive intakes of saturated fat [14] and red and/or processed meat [15]. Other dietary patterns, such as diets rich in whole fruits and vegetables [16], whole grains [17], dairy [18], and coffee [19], may potentially prevent or mitigate liver disease. Additionally, bacterial metabolites can contribute to liver disease by translocating to the liver where they act as ligands, binding to receptors that can either exacerbate or attenuate liver inflammation depending on the metabolite and other conditions, such as diet [20].

Given that diet influences the gut microbiome and metabolome [21], which may, in turn, modulate liver disease [22,23], a leading cause of morbidity and mortality in PLWH [3], we focus on diet quality among PLWH. Previous studies have reported diet quality to be poor in some PLWH population groups [24], and because diet is known to play a role even in the early stages of liver disease [25], exploring associations with diet may help to improve primary and secondary prevention efforts. Therefore, the purpose of this study was to explore associations between diet quality, the fecal microbiome, the metabolome, and liver health in PLWH from the Miami Adult Studies on HIV (MASH) cohort, which is comprised of mainly low-income, racial/ethnic minority adults that suffer high rates of substance use among other comorbidities.

## 2. Materials and Methods

In this cross-sectional study, an analysis was performed on 50 PLWH enrolled in a pilot study that aimed to characterize gut microbiota and bacterial metabolites in PLWH who use cocaine. The pilot study participants were randomly sampled from the MASH cohort. The participants were eligible if they had a confirmed HIV diagnosis, used cocaine or did not use any illicit drugs, were aged 35–66 years, were not infected with hepatitis C or hepatitis B, and had no antibiotic use during the three months prior to stool sample collection. All subjects provided written informed consent to participate. The protocols were approved by the Institutional Review Board at Florida International University.

Trained research staff administered 24-h dietary recalls to participants using the multiple-pass method [26]. The majority of participants had their fecal sample collected at their 18-month cohort visit, and we utilized all four 24-h recalls (one from every sixth-month cohort visit) to determine usual intake and calculate diet quality. The 24-h recalls were analyzed using NutriBase Pro software, version 17.2. (CyberSoft, Inc. Phoenix, AZ, USA). The United States Department of Agriculture (USDA) Healthy Eating Index (HEI)–2015, which has been validated as a reliable measure of diet quality [27], was used to measure diet quality. The USDA HEI uses a scoring system that ranges from 1 to 100, with higher HEI scores indicating better diet quality [28].

Stool samples from a single bowel movement were collected using the Norgen Biotek kit (Thorold, ON, Canada). Participants were instructed how to collect their fecal samples at home according to the manufacturer’s instructions; samples could be stored at room temperature. Participants were given their stool collection kit on the same cohort visit as their last 24-h dietary recall. This collection system has been shown to be effective in minimizing microbiota compositional shifts and may reduce bias in samples collected at ambient temperatures [29]. The samples were sent in one batch to the University of North Carolina (UNC) Microbiome Core overnight with ice packs.

The 16S ribosomal RNA (rRNA) gene amplicon sequencing was also conducted by the UNC Microbiome Core. All samples were processed within the same batch with the same lot of reagents. Amplification was performed using 12.5 nanograms of total DNA from the fecal samples, which was used to amplify the V4 region of the bacterial 16S rRNA gene using universal primers [30]. Each 16S rRNA gene amplicon was purified using the AMPURE XP reagent (Beckman Coulter, Indianapolis, Indiana, USA). A limited cycle Polymerase Chain Reaction program was applied to each sample. Illumina sequencing adapters and dual-index barcodes index 1 (i7) and index 2 (i5) (Illumina, San Diego, CA, USA) were added to the amplicon target. The final libraries were purified using AMPURE XP (Beckman Coulter) reagent and quantified and normalized before pooling. The libraries were denatured with NaOH, diluted with hybridization buffer, heat-denatured, and loaded on the MiSeq reagent cartridge (Illumina) and MiSeq instrument (Illumina). Paired-end sequencing with dual reads and automated cluster generation was completed according to the manufacturer’s instructions.

We obtained 200 µL of fasting blood plasma from the MASH cohort specimen repository that was obtained within three months of fecal sample collection and sent to Metabolon Inc. (Morrisville, NC, USA) for detection and analysis using metabolomics-non-targeted gas chromatography/mass spectrometry (GC-MS) and liquid chromatography/mass spectrometry (LC-MS).

Liver fibrosis was determined via the non-invasive FIB-4, calculated from age, se-rum aspartate aminotransferase (AST), serum alanine aminotransferase (ALT), and platelet count [31]. The negative predictive value of a FIB-4 < 1.45 cut-off to exclude advanced liver fibrosis was reported to be 90% with 70% sensitivity. A cut-off of FIB-4 > 3.25 had a positive predictive value of 65% for advanced liver fibrosis with 97% specificity [31]. However, only one participant in this pilot study had a FIB-4 > 3.25. Thus, we classified participants with FIB-4 < 1.45 as “advanced liver fibrosis/cirrhosis likely excluded”, and those with FIB-4 ≥ 1.45 as “unable to exclude advanced liver fibrosis/cirrhosis”.

Illumina Bcl2Fastq 2.18.0.12 was used to change the sequencing output from the Illumina MiSeq platform to the fastq format and demultiplex. QIIME 2 2018.11 was utilized to process the resulting paired-end reads [32]. The QIIME 2 invocation of cutadapt was used to trim the index and linker primer sequences. DADA2 through QIIME 2 was used to process the resulting paired end reads. Taxonomic identifiers with respect to Green Genes release 13_08 were assigned to the amplicon sequencing units from DADA2. The read counts of amplicon sequence variants (ASVs) were retrieved from QIIME 2, aggregated at the genus level, and normalized for each sample to adapt to one.

Linear discriminant analysis effect size (LefSe) was performed to assess the differential abundance of bacterial taxa. Phyloseq R package [33], was utilized to perform permutational multivariate analysis of variance (PERMANOVA) with Unifrac distances [34]. Unifrac distance matrix was computed with phyloseq R package [33], while PER-MANOVA analysis was performed with vegan R library [35]. Metabolomics analysis data processing included data imputation of missing values using k-nearest neighbor (kNN) imputation [36], outlier detection via kNN clustering, normalization with log2 scaling, and quality control using kNN and variance stabilizing normalization (VSN) combined [37]. Wilcoxon rank-sum tests were used to determine statistical significance between the variables. The proportionality between effect size and statistical significance was explored with volcano plots. Pathway detection and differential analysis were performed with MetaboDiff R package [38]. Partial least squares–discriminant analysis (PLS-DA) was used to find discriminative metabolites [39].

Demographic data (age, sex, race, ethnicity, education level, income, food insecurity) were self-reported. Alcohol and tobacco use were self-reported, while substance use was determined with urine toxicology. Anthropometric data, including height and weight, were measured by trained research staff. HIV serostatus and years living with HIV were obtained by self-report and confirmed with medical documentation. HIV viral load and cluster of differentiation 4 (CD4) cell count were abstracted from medical records with participants’ written approval and ART use, regimen, and adherence were self-reported by participants.

Descriptive continuous variables are presented as mean ± SD and categorical variables are presented as No. (%). To test for differences in demographics, *t*-tests were performed for continuous variables and chi-squared tests were employed for categorical variables. Fisher’s exact test was utilized in cases of small cell counts. The mean total HEI score for the sample was compared to the mean HEI score for Americans as reported by the National Center for Health Statistics [40], using a one sample *t*-test. Spearman’s correlation was used to test for associations between HEI scores and FIB-4 scores. *t*-tests were utilized to test for differences in mean HEI score by FIB-4 score. Results were considered statistically significant at two-tailed *p* < 0.05. All statistical analyses were performed using SAS software, Version 9.4 (SAS, Inc., Cary, NC, USA).

## 3. Results

### 3.1. Demographics

The characteristics of the study sample are presented in Table 1. The average age was 55 ± 6.8 years, 58% were male, 68% were non-Hispanic Black, 40% had obtained less than a high school level education, 68% had an annual income of less than USD 12,500, and 98% were on ART. Participants with and without FIB-4 ≥ 1.45 were similar in age, sex, race/ethnicity, education level, income, smoking, alcohol use, cocaine use, body mass index (BMI), food insecurity, ART use and adherence, viral load, and years living with HIV. There were also no significant differences between participants with and without FIB-4 ≥ 1.45 by ART regimen. However, the mean CD4 cell count was significantly lower in participants with FIB-4 ≥ 1.45 compared to participants with FIB-4 < 1.45 (*p* = 0.007).

### 3.2. Diet Quality in PLWH

The mean total HEI score of the sample (45.67 ± 11.54) was significantly lower (*p* < 0.0001) compared to the HEI score for Americans (μ = 59) [40]. Table 2 shows dietary intake and dietary quality as measured by the HEI. On average, participants had low HEI component scores for total fruit (2.16 ± 1.80), whole fruit (1.48 ± 1.94), total vegetables (2.53 ± 1.43), whole grains (2.80 ± 3.25), dairy (2.32 ± 1.80), and mono- and polyunsaturated fats (0.27 ± 1.01), indicating low intake of these foods. The participants also had low average HEI component scores for saturated fat (6.37 ± 3.04), refined grains (4.22 ± 3.62), sodium (3.93 ± 3.46), and added sugar (6.39 ± 2.90), indicating high intake of these foods.

### 3.3. Diet Quality and FIB-4

Compared to participants with FIB-4 ≥ 1.45, participants with FIB-4 < 1.45 had significantly higher intake of dairy (*p* = 0.006) and dairy HEI component scores (*p* = 0.036) (Table 2). Using Spearman’s correlation, FIB-4 score was significantly inversely correlated with dairy HEI component score (*r* = −0.31, *p* = 0.027). A significant inverse correlation was also observed between FIB-4 score and seafood and plant protein HEI component score (*r* = −0.320, *p* = 0.022). There were no significant differences found in total HEI score between participants with and without FIB-4 ≥ 1.45, and there were no correlations found between total HEI score and FIB-4 score using Spearman’s correlation.

### 3.4. Fecal Microbial Taxa by FIB-4 Score

**Figure 1:**
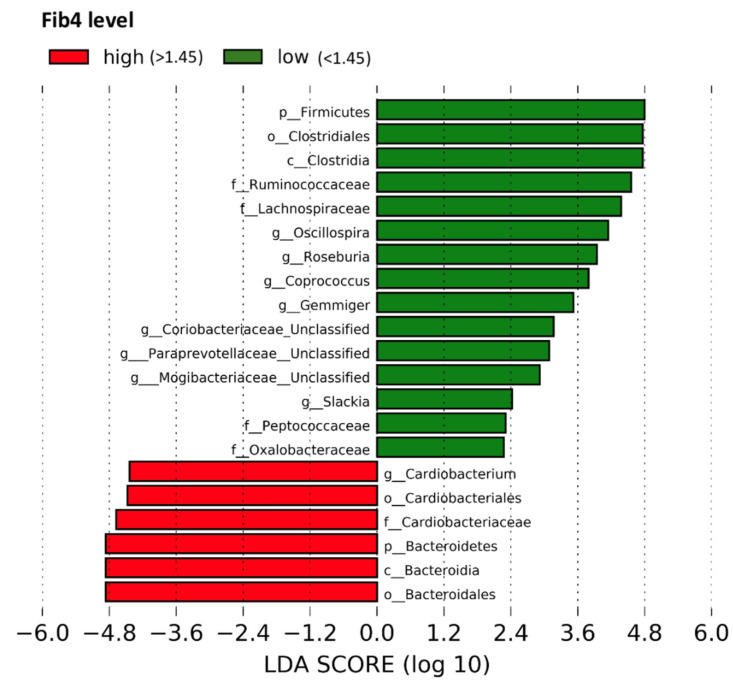
Differential relative abundance of participants with and without FIB-4 ≥ 1.45. Figure 1 displays the results of the LEfSe analysis, which identifies the most differentially abundant taxa between participants with and without FIB-4 ≥ 1.45. FIB-4 score ≥ 1.45 was defined as “unable to exclude advanced liver fibrosis/cirrhosis”. Taxa enriched in participants with FIB-4 < 1.45 are indicated with a positive LDA score in green, and taxa enriched participants with FIB-4 ≥ 1.45 are indicated with a negative score in red. Abbreviations: FIB-4, Fibrosis-4 Index; LDA, linear discriminant analysis; LEfSe, linear discriminant analysis effect size.

By utilizing LefSe, this study found that the relative abundances of butyrate-producing taxa such as *Ruminococcaceae*, *Roseburia* species (spp.), and *Lachnospiraceae* were higher in participants with FIB-4 < 1.45. The relative abundances of the Firmicutes phylum, Clostridiales order, Clostridia class, *Peptococcaceae*, *Coriobacteriaceae*, *Paraprevotellaceae*, *Mogibacteriaceae*, and *Oxalobacteraceae* families, and the genera *Oscillospira*, *Coprococcus*, *Gemmiger*, and *Slackia* were also higher in participants with FIB-4 < 1.45. On the other hand, the relative abundances of the Bacteroidales and Cardiobacteriales orders, Bacteroidia class, Bacteroidetes phylum, *Cardiobacteriaceae* family, and *Cardiobacterium* genus were higher in those with FIB-4 ≥ 1.45 (Figure 1). Using PERMANOVA, however, the variance in metagenomics was not significantly different between participants with and without FIB-4 ≥ 1.45 (*p* = 0.671) (Table A1).

### 3.5. Microbial Metabolites by FIB-4 Score

**Figure 2:**
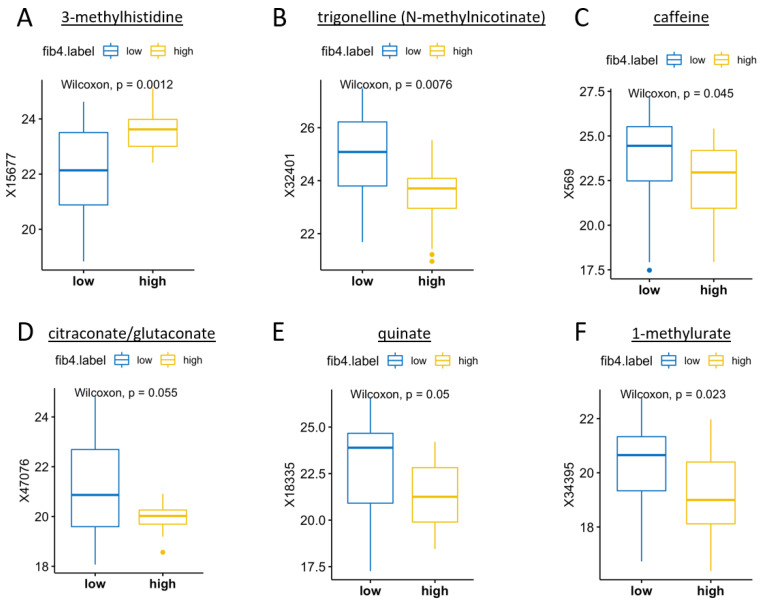
The most differentially expressed metabolites in participants with and without FIB-4 ≥ 1.45. Figure 2 displays metabolites that differed significantly in participants with and without FIB-4 ≥ 1.45 determined by *p* < 0.05 in Wilcoxon test. FIB-4 score ≥ 1.45 (“high” FIB-4 label) was defined as “unable to exclude advanced liver fibrosis/cirrhosis”. (**A**) Methylhistidine, beta-alanine, and histidine metabolism; (**B**) niacin metabolism and biomarker for consumption of coffee, legumes and soy products; (**C**) biomarker of caffeine consumption; (**D**) methyl-branched fatty acids involved in lipid transport and metabolism; (**E**) a crystalline acid obtained from cinchona bark, coffee beans, carrot leaves, apples, peaches, vegetables, etc., that is implicated in the perceived acidity of coffee; (**F**) the product of the metabolism of methylxanthines (caffeine, theophylline, and theobromine). Abbreviations: FIB-4, Fibrosis-4 Index.

**Figure 3:**
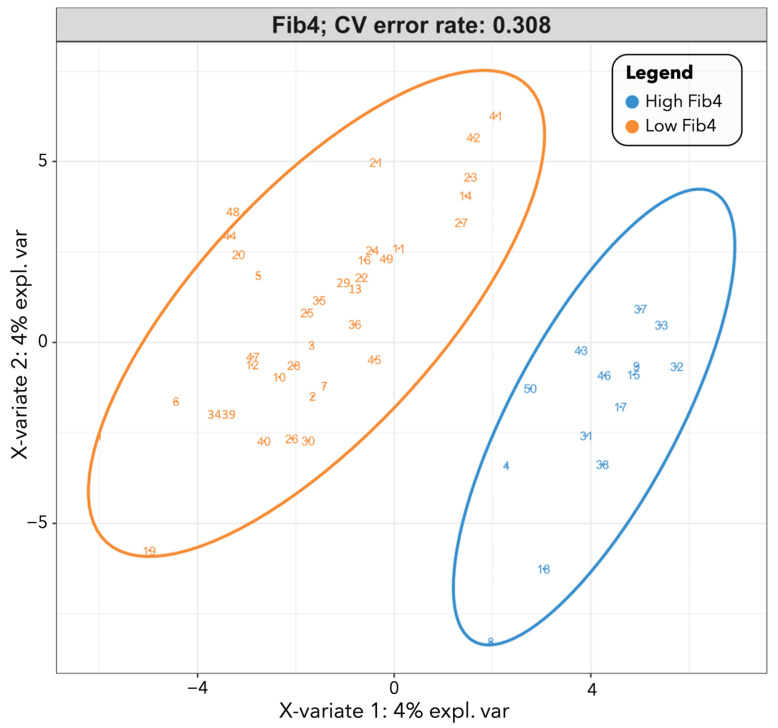
PLS-DA score plot for participants with and without FIB-4 ≥ 1.45. Figure 3 displays the class prediction plot representing bacterial metabolites separating participants with and without FIB-4 ≥ 1.45 the most based on the first two principal components of the PLS-DA model. FIB-4 score ≥ 1.45 (“high FIB-4” label) was defined as “unable to exclude advanced liver fibrosis/cirrhosis”. Each point on the graph represents a participant, with blue circles representing participants with FIB-4 ≥ 1.45 and orange triangles representing participants with FIB-4 < 1.45. Abbreviation: PLS-DA, partial least squares–discriminant analysis.

**Figure A1:**
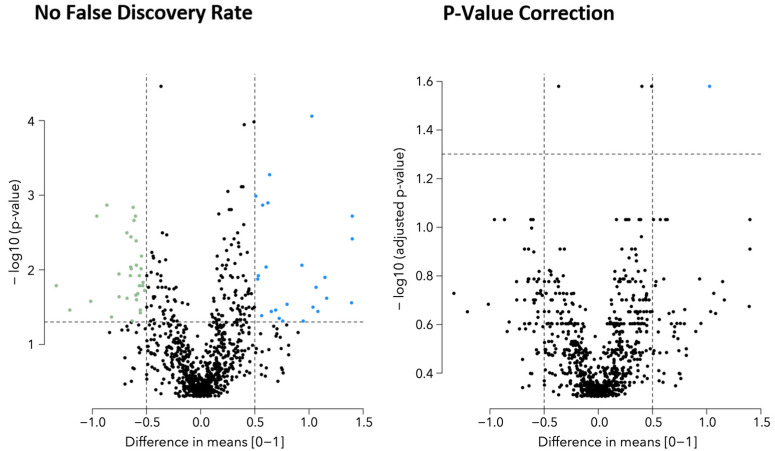
Volcano plots to identify microbial metabolites that significantly differed between participants with and without FIB-4 ≥ 1.45. Figure A1 displays the volcano plots that were used to determine statistically significant metabolites that had an absolute difference in means (dm) >0.5 between those with and without FIB-4 ≥ 1.45. The X-axis shows the difference in means of the concentrations of the metabolites after the variance-stabilizing normalization, while the Y-axis shows a negative log of p-value determined by Wilcoxon rank-sum tests without false discovery rate (**left plot**) and with Benjamini–Hochberg *p*-value correction (**right plot**). Blue points (upper right) show metabolites that have significantly higher concentrations in the cohort with FIB-4 ≥ 1.45 (dm > 0.5, *p*-value < 0.05), while green points (upper left) show metabolites that have significantly higher concentrations in the group with FIB-4 < 1.45 (dm < −0.05, *p*-value < 0.05). The two vertical dotted lines represent the two thresholds showing dm >0.5, while the dotted horizontal line represents a p-value threshold of 0.05 (i.e., negative log *p*-value of 1.3). FIB-4 score ≥ 1.45 was defined as “unable to exclude advanced liver fibrosis/cirrhosis”. Abbreviation: FIB-4, Fibrosis-4 Index.

Participants with FIB-4 < 1.45 had significantly higher levels of caffeine (*p* = 0.045) and related metabolites trigonelline (*p* = 0.008) and 1-methylurate (*p* = 0.023). Participants with FIB-4 ≥ 1.45 had significantly higher levels of 3-methylhistidine (*p* = 0.001), a metabolite associated with chronic diseases such as obesity and type II diabetes (Figure 2). Figure 3 displays the class prediction plot based on the first two principal components of the PLS-DA and shows separate clustering of participants with and without FIB-4 ≥ 1.45 (CV error = 0.308). We reported microbial metabolites that were significantly different between participants with and without FIB-4 ≥ 1.45 (as determined by Wilcoxon test), with an absolute difference >0.5 determined via volcano plot (Figure A1), as contributing most to class separation by PLS-DA, and correlated with FIB-4 score (Table 3). Several microbial metabolites related to lipid pathways including pregnen-diol disulfate (*r* = 0.333, *p* = 0.018), 9,10-dihydroxy-12-octadecenoic acid (9,10-diHOME) (*r* = 0.454, *p* = 0.0009), and octadecanedioylcarnitine (*r* = 0.375, *p* = 0.007) were significantly correlated with FIB-4 ≥ 1.45 (Table 3).

## 4. Discussion

This analysis aimed to explore associations between diet quality, the fecal microbiome, the metabolome, and liver health in PLWH with and without FIB-4 ≥ 1.45 (n = 14 vs. n = 36, respectively). The participants in this study displayed poorer diet quality compared to the general U.S. population [40]. While the participants had similar sociodemographic, substance abuse, and HIV characteristics, except for CD4 cell count, which was lower in participants with FIB-4 ≥ 1.45, those with FIB-4 < 1.45 had higher intakes of dairy, and we observed an inverse correlation between FIB-4 and seafood and plant protein HEI component score. We did not find a significant relationship between total HEI score and FIB-4, suggesting specific dietary components may be a better target for dietary interventions aimed to prevent liver disease in PLWH. Participants with FIB-4 < 1.45 had higher relative abundances of butyrate-producing taxa, as well as greater amounts of caffeine and related metabolites. These findings are relevant to low-income, marginalized populations of PLWH, who tend to have poor quality diets [24], and increased morbidity and mortality due to liver disease and other comorbidities [3]. Improving diet quality in vulnerable PLWH by targeting specific dietary components may help to simplify and personalize dietary interventions to prevent the development or progression of liver disease.

Few studies have examined diet quality of PLWH in the U.S. using the HEI. Our study is novel in that we assessed diet quality in a sample of low-income, racial/ethnic-minority PLWH by utilizing multiple 24-hour recalls collected over 18 months to calculate HEI scores. Weiss et al. investigated diet quality in PLWH using the HEI and reported poorer diet quality in PLWH compared to HIV-negative participants including significantly lower seafood, plant protein, and beneficial fatty acid consumption [24]. Our study concurs with Weiss et al. in that our sample of underserved PLWH had poorer diet quality compared to the general U.S. population [40]. However, our sample of low-income PLWH had poorer diet quality, with an average HEI score of 45.7, than those in Weiss et al., which had an average total HEI score of 51.3 [24]. Volpe et al. also used the HEI to assess diet quality in PLWH [41]; however, the average total HEI score of our sample of vulnerable PLWH was, again, much lower (45.7 vs. 61.7) [41]. This could be due to differences in eligibility criteria, as Volpe et al. excluded obese participants [41].

Fibrosis-4 index score was weakly inversely correlated with intake of dairy. Our findings in PLWH might be explained by Kratz et al., who showed dairy fat intake was associated with improved glucose tolerance, hepatic insulin sensitivity, and reduced liver fat in a sample of adults living without HIV [18]. The potential hepatoprotective role of dairy consumption may be due to specific components of dairy, such as the trans-palmitoleate present in dairy fat described by Kratz et al. [18]. Our study also found an inverse correlation between FIB-4 score and seafood and plant protein intake. If confirmed, it could be recommended that PLWH consume more dairy products and replace processed red meat with seafood and plant protein, being that excessive saturated fat and red/processed meat intake have been associated with liver disease [15]. Research into federal programs and policy should be considered to understand the feasibility of these recommendations for underserved and vulnerable populations.

This study did not find any relationships between total HEI score and FIB-4 score. This could be due to the small sample size of the study and unequal distribution of participants with and without FIB-4 ≥ 1.45 (n = 14 vs. n = 36, respectively), making it difficult to detect differences in total HEI score, which is comprised of 13 different HEI component scores. Additionally, the MASH cohort, the source of the recruited participants, is composed mainly of low-income, racial/ethnic-minority adults that suffer high rates of substance use and food insecurity [42]. This may have contributed to the low total HEI scores seen in both participants with and without FIB-4 ≥ 1.45. These findings are in agreement with Weiss et al., who also did not find statistically significant differences in total HEI score, but instead found significant differences in multiple HEI component scores [24].

Participants with FIB-4 < 1.45 had higher relative abundances of beneficial, butyrate-producing taxa *Ruminococcaceae, Roseburia*, and *Lachnospiraceae*. Butyrate, a short-chain fatty acid (SCFA), is an energy source for enterocytes and helps to maintain gut barrier function [43]. Potentially due to their role in the maintenance of gut barrier integrity, SCFA have been shown to have a hepatoprotective effect [44]. Previous studies have reported significant decreases in these taxa in cirrhotic patients [45]. Liu et al. found these taxa to be associated with dietary quality using the HEI [46]. Furthermore, LPS, a marker of bacterial translocation that is known to upregulate inflammatory cells in the liver via the gut–liver axis [4], has been reported to be negatively associated with these taxa [45]. Taken together, these findings indicate that beneficial microbiota, such as those that produce butyrate and other SCFA, may be important in maintaining gut barrier integrity and preventing translocation of pathogenic microbial products to the liver via the gut–liver axis, which may prevent or attenuate liver fibrosis in PLWH [20].

We reported higher relative abundances of Firmicutes in participants with FIB-4 < 1.45, which concurs with the gut microbiome metagenomic signature reported by Loomba et al., which distinguished mild/moderate liver fibrosis from advanced fibrosis [11]. However, this study focused on PLWH and considered dietary quality which differs from Loomba et al. [11]. On the other hand, in participants with FIB-4 ≥ 1.45, we found higher relative abundances of the Bacteroidetes phylum and Bacteroidales order. Bacteroidales have been associated with alcoholic liver disease in mice [47]. The Bacteroidetes phylum, however, has been reported previously to be less abundant in NAFLD [22] and cirrhosis [10], but enriched in nonalcoholic steatohepatitis (NASH) [23]. This suggests that different stages of liver disease may present with important differences in gut microbiome composition. Despite finding higher relative abundances of butyrate-producing bacterial taxa in participants with FIB-4 < 1.45 and some potentially pathogenic bacterial taxa in those with FIB-4 ≥ 1.45, we did not find significant differences in the variance of metagenomics between participants with and without FIB-4 ≥ 1.45. This could be due to the small sample size, unequal distribution of participants with and without FIB-4 ≥ 1.45, and use of a non-direct estimation of liver fibrosis that classified participants as either FIB-4 < 1.45 (advanced liver fibrosis/cirrhosis likely excluded) or FIB-4 ≥ 1.45 (unable to exclude advanced liver fibrosis/cirrhosis). Given that previous studies have reported differences in microbial composition by liver fibrosis severity [11,12], this could have contributed to the lack of variance in metagenomics seen. Also worth noting is the possibility of ART interruption influencing liver health due to an increase in HIV viral load [48,49]. We did find that participants with FIB-4 ≥ 1.45 had a lower mean CD4 cell count, but there were no significant differences in the proportions of participants who were virally suppressed or the mean number of missed ART doses in the previous two weeks in participants with and without FIB-4 ≥ 1.45.

In this study, caffeine and related metabolites trigonelline and 1-methylurate were found in greater amounts in participants with FIB-4 < 1.45. Caffeine is most commonly consumed in coffee, and a recent study reported that coffee consumption significantly lowers the risk of NAFLD and hepatocellular carcinoma, potentially due to active chemical compounds present in coffee such as caffeine [19]. Caffeine has been reported to be anti-inflammatory in nature and has demonstrated immunomodulatory effects that occur in amounts relevant to normal human consumption [50]. The results of this study indicate that caffeine may be partly responsible for the health benefits of regular coffee consumption.

Participants with FIB-4 ≥ 1.45 had higher levels of 3-methylhistidine, a metabolite that has been associated with obesity and type II diabetes [51]. A FIB-4 ≥ 1.45 was also correlated with several microbial metabolites related to lipid pathways, including pregnen-diol disulfate, 9,10-diHOME, and octadecanedioylcarnitine. Alterations in lipid metabolism have been described in NAFLD, alcoholic liver disease, cirrhosis, and viral hepatitis [52]. Further research is needed to define the role these metabolites may have in liver disease development and progression.

## 5. Conclusions

We present the first study aimed at exploring associations between diet quality, the fecal microbiome, the metabolome, and liver health in a sample of low-income, underserved, racial/ethnic-minority, vulnerable PLWH. Strengths of this study include the use of a multi-omics approach, validated measure of diet quality, and recruitment of participants from the MASH cohort which allowed for utilization of multiple 24-h recalls collected over 18 months, leading to a more comprehensive assessment of diet quality. However, the limitations of this study should be noted. Namely, the small sample size, the unequal distribution of participants with and without FIB-4 ≥ 1.45, the cross-sectional design, which does not allow for temporality or causality to be established, and the use of the FIB-4, an indirect measure of liver fibrosis. Additionally, lack of a control group consisting of participants without HIV precluded the ability to explore the relationship of HIV itself with the variables of interest. Nevertheless, our findings provide preliminary evidence that specific dietary components, such as dairy, caffeine, seafood, and plant protein may be associated with liver health by way of the microbiome and metabolome, and if confirmed, may potentially be targeted in dietary interventions aimed to reduce liver-related morbidity and mortality in underserved and vulnerable PLWH. Future randomized controlled trials that incorporate multi-omics approaches are needed to fully understand the relationships between diet quality, the intestinal microbiome, the metabolome, and liver fibrosis in PLWH.

## Figures and Tables

**Table 1:** Characteristics of Study Participants.

Variable ^1^	Total n = 50	FIB-4 < 1.45 n = 36	FIB-4 ≥ 1.45 ^2^ n = 14	*p*
Age, years	55 ± 6.8	54.5 ± 7.5	56.2 ± 4.6	0.437
Sex, male	29 (58.0)	19 (52.8)	10 (71.4)	0.341
Race/ethnicity				0.538
White non-Hispanic	3 (6.0)	3 (8.3)	0	
Black non-Hispanic	34 (68.0)	22 (61.1)	12 (85.7)	
White Hispanic	12 (24.0)	10 (27.8)	2 (14.3)	
Other	1 (2.0)	1 (2.8)	0	
Education level				
<High school	20 (40.0)	17 (47.2)	3 (21.4)	0.227
High school diploma/GED	18 (36.0)	12 (33.3)	6 (42.9)	
Some college +	12 (24.0)	7 (19.4)	5 (35.7)	
Annual income				0.525
<USD 12,500	34 (68.0)	24 (66.7)	10 (71.4)	
USD 12,500–USD 35,000	14 (28.0)	11 (30.6)	3 (21.4)	
≥USD 35,000	2 (4.0)	1 (2.8)	1 (7.1)	
Tobacco use	26 (52.0)	17 (47.2)	9 (64.3)	0.196
Non-smoker	24 (48.0)	19 (52.8)	5 (35.7)	
Every day smoker	13 (26)	10 (27.8)	3 (21.4)	
Some days smoker	13 (26)	7 (19.4)	6 (42.9)	
Hazardous alcohol use ^3^	7 (14.0)	5 (13.9)	2 (14.3)	1.000
Cocaine use	25 (50.0)	17 (47.2)	8 (57.1)	0.754
Obesity ^4^	20 (40.0)	16 (44.4)	4 (28.6)	0.445
Food insecurity ^5^	6 (12.0)	3 (8.3)	3 (21.4)	0.331
On ART	49 (98.0)	36 (100.0)	13 (92.9)	0.280
ART regimen				
Multi-class combination products	46 (92.0)	35 (97.2)	11 (78.6)	0.061
NRTI	0	0	0	-
NNRTI	1 (2.0)	0	1 (7.1)	0.280
Protease inhibitors	7 (14.0)	4 (11.1)	3 (21.4)	0.384
Fusion inhibitors	0	0	0	-
Entry inhibitors	0	0	0	-
HIV integrase strand transfer inhibitors	5 (10.0)	3 (8.3)	2 (14.3)	0.611
Pharmacokinetic enhancers	0	0	0	-
Post-attachment inhibitor	0	0	0	-
ART adherence ^6^	0.53 ± 1.3	0.41 ± 1.1	0.85 ± 1.7	0.316
Virally suppressed ^7^	34 (68.0)	27 (75.0)	7 (50.0)	0.105
CD4 lymphocyte count, cells/µL	634.6 ± 337.1	694.9 ± 367.0	479.4 ± 172.3	0.007 *
Years living with HIV	17.6 ± 8.2	17.5 ± 7.8	17.8 ± 9.4	0.922

^1^ Data are presented as mean ± standard deviation and n (%). ^2^ FIB-4 score ≥ 1.45 was defined as “unable to exclude advanced liver fibrosis/cirrhosis”. ^3^ Hazardous alcohol use was defined as having a score of ≥4 for men and ≥3 for women on the Alcohol Use Disorder Identification Test-Consumption (AUDIT-C) Questionnaire. ^4^ Obesity was defined as having a body mass index (BMI) of ≥30 kg/m^2^. ^5^ Food insecurity was defined as having a score of ≥3 on the USDA Household Food Security Survey Module. ^6^ ART adherence is reported as the mean number of missed ART doses in the previous two weeks. ^7^ Virally suppressed was defined as <50 copies of HIV per mL of blood. * *p*-value < 0.05. Abbreviations: ART, antiretroviral therapy; CD4, cluster of differentiation 4; FIB-4, Fibrosis-4 Index; NNRTI, non-nucleoside reverse transcriptase inhibitor; NRTI, nucleoside/nucleotide reverse transcriptase inhibitor.

**Table 2:** Dietary intake and HEI scores of study participants by FIB-4 Score.

Variable ^1^	Max Possible Score	Total n = 50	FIB-4 < 1.45 n = 36	FIB-4 ≥ 1.45 ^2^ n = 14	*p*
Total caloric intake (kcal)	–	2087 ± 757	2200 ± 804	1795 ± 540	0.089
Adequacy components:					
Total fruit HEI score ^3^	5	2.16 ± 1.80	2.13 ± 1.86	2.23 ± 1.70	0.872
Total fruit intake (cups)	–	1.01 ± 1.35	1.13 ± 1.55	0.69 ± 0.56	0.142
Whole fruit HEI score ^4^	5	1.48 ± 1.94	1.50 ± 1.89	1.45 ± 2.14	0.930
Whole fruit intake (cups)	–	0.51 ± 0.98	0.60 ± 1.10	0.28 ± 0.49	0.167
Total vegetable HEI score ^5^	5	2.53 ± 1.43	2.64 ± 1.50	2.23 ± 1.22	0.370
Total vegetable intake (cups)	–	1.22 ± 0.91	1.31 ± 0.91	1.00 ± 0.91	0.292
Greens & beans HEI score ^5^	5	4.05 ± 1.56	4.23 ± 1.46	3.60 ± 1.79	0.205
Greens & beans (cups)	–	0.66 ± 0.60	0.74 ± 0.62	0.47 ± 0.52	0.157
Whole grains HEI score	10	2.80 ± 3.25	2.61 ± 3.30	3.26 ± 3.18	0.533
Whole grains intake (oz)	–	1.00 ± 1.47	0.95 ± 1.45	1.12 ± 1.56	0.720
Dairy HEI score ^6^	10	2.32 ± 1.80	2.65 ± 1.77	1.47 ± 1.65	0.036 *
Dairy intake (cups)	–	0.61 ± 0.47	0.73 ± 0.48	0.33 ± 0.29	0.006 *
Total protein foods HEI score ^5^	5	5.00 ± 0.00	5.00 ± 0.00	5.00 ± 0.00	1.000
Protein intake (oz)	–	10.90 ± 4.45	10.74 ± 4.52	11.34 ± 4.41	0.672
Seafood and plant proteins HEI score ^5,7^	5	4.15 ± 1.44	4.31 ± 1.35	3.74 ± 1.63	0.214
Seafood/plant protein intake (oz)	–	3.03 ± 2.45	3.35 ± 2.61	2.21 ± 1.82	0.141
Fatty acids HEI score ^8^	10	0.27 ± 1.01	0.24 ± 1.02	0.37 ± 1.00	0.688
Polyunsaturated fatty acid intake (g)	–	4.84 ± 5.41	5.17 ± 5.94	3.99 ± 3.78	0.493
Monounsaturated fatty acid intake (g)	–	6.87 ± 6.19	7.51 ± 6.85	5.21 ± 3.72	0.135
Moderation components:					
Saturated fatty acid HEI score	10	6.37 ± 3.04	6.43 ± 2.88	6.21 ± 3.53	0.822
Saturated fatty acid intake (g)	–	25.72 ± 13.33	27.00 ± 13.48	22.41 ± 12.82	0.278
Refined grains HEI score	10	4.22 ± 3.62	4.19 ± 3.63	4.28 ± 3.72	0.939
Refined grains intake (oz)	–	7.37 ± 4.40	7.66 ± 4.52	6.63 ± 4.16	0.462
Sodium HEI score	10	3.93 ± 3.46	3.81 ± 3.45	4.24 ± 3.61	0.694
Sodium intake (g)	–	3.48 ± 1.16	3.63 ± 1.09	3.10 ± 1.30	0.151
Added sugar HEI score	10	6.39 ± 2.90	6.34 ± 3.05	6.52 ± 2.59	0.845
Added sugar intake (% energy)	–	0.13 ± 0.06	0.14 ± 0.07	0.13 ± 0.06	0.775
HEI total score ^9^	100	45.67 ± 11.54	46.08 ± 11.52	44.60 ± 11.96	0.689

^1^ Values are presented as mean ± SD. ^2^ FIB-4 score ≥ 1.45 was defined as “unable to exclude advanced liver fibrosis/cirrhosis”. ^3^ Includes 100% fruit juice. ^4^ Includes all forms except juice. ^5^ Includes legumes (beans and peas). ^6^ Includes all milk products (milk, yogurt, cheese, and fortified soy beverages since these are nutritionally similar to dairy products). ^7^ Includes seafood, nuts, seeds, soy products (other than beverages), and legumes (beans and peas). ^8^ Ratio of poly- and monounsaturated fatty acids to saturated fatty acids. ^9^ All scoring standards were adopted from “United States Department of Agriculture: Food and Nutrition Service: Center for Nutrition Policy and Promotion (CNPP). How the HEI Is Scored”; https://www.fns.usda.gov/how-hei-scored (accessed 30 December 2020). * *p*-value < 0.05. Abbreviations: FIB-4, Fibrosis-4 Index; HEI, Healthy Eating Index.

**Table 3:** Microbial metabolites that significantly differentiated participants with and without FIB-4 ≥ 1.45. ^1^

Biochemical	Super Pathway	Sub Pathway	Metabolite	*PLS-DA Effect Size*	*Spearman r*	*Spearman p-Value*
Pregnen-diol disulfate *	Lipid	Pregnenolone steroids	32,562	0.249	0.333	0.018
1-stearoyl-GPS (18:0) *	Lipid	Lysophospholipid	45,966	−0.253	−0.283	0.047
1,3-dimethylurate	Xenobiotics	Xanthine metabolism	32,391	−0.090	−0.292	0.040
2-deoxyuridine	Nucleotide	Pyrimidine metabolism, uracil containing	52,602	0.262	0.344	0.015
Serotonin	Amino acid	Tryptophan metabolism	2342	−0.059	−0.316	0.025
Inosine 5-monophosphate (IMP)	Nucleotide	Purine metabolism, (hypo)xanthine/inosine containing	2133	−0.174	−0.396	0.004
1-stearoyl-2-oleoyl-GPS (18:0/18:1)	Lipid	Phosphatidylserine (PS)	19265	−0.192	−0.391	0.005
Cysteine-glutathione disulfide	Amino acid	Glutathione metabolism	35,159	−0.074	−0.288	0.042
2-hydroxy-4-(methylthio)butanoic acid	Amino acid	Methionine, cysteine, SAM, and taurine metabolism	63,739	0.109	0.299	0.035
AMP	Nucleotide	Purine metabolism, adenine containing	32,342	−0.204	−0.503	0.0002
9,10-diHOME	Lipid	Fatty acid, dihydroxy	38,399	0.213	0.454	0.001
6-oxopiperidine-2-carboxylate	Amino acid	Lysine metabolism	43,231	0.227	0.280	0.049
Octadecanedioylcarnitine (C18-DC) *	Lipid	Fatty acid metabolism (Acyl carnitine, dicarboxylate)	61,867	0.134	0.375	0.007
S-methylcysteine sulfoxide	Amino acid	Methionine, cysteine, SAM, and taurine metabolism	43,378	−0.004	−0.317	0.025

Table 3 displays microbial metabolites that satisfied the following conditions: (1) they were significantly different between participants with and without FIB-4 ≥ 1.45 (as determined by Wilcoxon test); (2) they had an absolute difference >0.5 determined via volcano plot; (3) they were found to contribute most to class separation by PLS-DA; and (4) they correlated with FIB-4 score via Spearman’s correlation. ^1^ FIB-4 score ≥ 1.45 was defined as “unable to exclude advanced liver fibrosis/cirrhosis”. Rows in light grey indicate FIB-4 < 1.45 and rows in dark grey indicate FIB-4 ≥ 1.45. * Indicates a compound that has not been confirmed based on a standard, but Metabolon Inc. is confident in its identity. Abbreviation: PLS-DA, partial least squares-discriminant analysis.

## Data Availability

The data presented in this study are available on request from the corresponding authors. The data are not publicly available due to privacy and ethical restrictions.

## Appendix A

**Table A1:** Permutational multivariate analysis of variance (PERMANOVA) metagenomics analysis (UniFrac similarity).

	d.f.	Sum of Squares	Mean Square	F	R^2^	*p*-Value
FIB-4 ≥ 1.45 ^1^	1	0.3375	0.3375	0.9446	0.0192	0.671

Table A1 displays the results of the PERMANOVA analysis. The variance in metagenomics between participants with and without FIB-4 ≥ 1.45 was not significantly different. ^1^ FIB-4 score ≥ 1.45 was defined as “unable to exclude advanced liver fibrosis/cirrhosis”. Abbreviation: FIB-4, Fibrosis-4 Index; PERMANOVA, permutational multivariate analysis of variance.

## References

[1] May M.T., Gompels M., Delpech V., Porter K., Orkin C., Kegg S., Hay P., Johnson M., Palfreeman A., Gilson R. (2014). Impact on life expectancy of HIV-1 positive individuals of CD4+ cell count and viral load response to antiretroviral therapy. Aids.

[2] Farahani M., Mulinder H., Farahani A., Marlink R. (2017). Prevalence and distribution of non-AIDS causes of death among HIV-infected individuals receiving antiretroviral therapy: A systematic review and meta-analysis. Int. J. STD AIDS.

[3] Croxford S., Kitching A., Desai S., Kall M., Edelstein M., Skingsley A., Burns F., Copas A., Brown A.E., Sullivan A.K. (2017). Mortality and causes of death in people diagnosed with HIV in the era of highly active antiretroviral therapy compared with the general population: An analysis of a national observational cohort. Lancet Public Health.

[4] Kaspar M.B., Sterling R.K. (2017). Mechanisms of liver disease in patients infected with HIV. BMJ Open Gastroenterol..

[5] Sherman K.E., Rockstroh J., Thomas D. (2015). Human immunodeficiency virus and liver disease: An update. Hepatology.

[6] Tamargo J.A., Sherman K.E., Sékaly R.P., Bordi R., Schlatzer D., Lai S., Khalsa J.H., Mandler R.N., Ehman R.L., Baum M.K. (2022). Cocaethylene, simultaneous alcohol and cocaine use, and liver fibrosis in people living with and without HIV. Drug Alcohol Depend..

[7] Iacob S., Iacob D.G. (2019). Infectious threats, the intestinal barrier, and its Trojan Horse: Dysbiosis. Front. Microbiol..

[8] Kim C.J., McKinnon L.R., Kovacs C., Kandel G., Huibner S., Chege D., Shahabi K., Benko E., Loutfy M., Ostrowski M. (2013). Mucosal Th17 cell function is altered during HIV infection and is an independent predictor of systemic immune activation. J. Immunol..

[9] Serrano-Villar S., Rojo D., Martínez-Martínez M., Deusch S., Vázquez-Castellanos J.F., Bargiela R., Sainz T., Vera M., Moreno S., Estrada V. (2016). Gut bacteria metabolism impacts immune recovery in HIV-infected individuals. EBioMedicine.

[10] Chen Y., Yang F., Lu H., Wang B., Lei D., Wang Y., Zhu B., Li L. (2011). Characterization of fecal microbial communities in patients with liver cirrhosis. Hepatology.

[11] Loomba R., Seguritan V., Li W., Long T., Klitgord N., Bhatt A., Dulai P.S., Caussy C., Bettencourt R., Highlander S.K. (2017). Gut microbiome-based metagenomic signature for non-invasive detection of advanced fibrosis in human nonalcoholic fatty liver disease. Cell Metab..

[12] Lang S., Farowski F., Martin A., Wisplinghoff H., Vehreschild M.J.G.T., Krawczyk M., Nowag A., Kretzschmar A., Scholz C., Kasper P. (2020). Prediction of advanced fibrosis in non-alcoholic fatty liver disease using gut microbiota-based approaches compared with simple non-invasive tools. Sci. Rep..

[13] Humphreys C. (2020). Intestinal Permeability. Textbook of Natural Medicine.

[14] Luukkonen P.K., Sädevirta S., Zhou Y., Kayser B., Ali A., Ahonen L., Lallukka S., Pelloux V., Gaggini M., Jian C. (2018). Saturated fat is more metabolically harmful for the human liver than unsaturated fat or simple sugars. Diabetes Care.

[15] Zelber-Sagi S., Ivancovsky-Wajcman D., Fliss Isakov N., Webb M., Orenstein D., Shibolet O., Kariv R. (2018). High red and processed meat consumption is associated with non-alcoholic fatty liver disease and insulin resistance. J. Hepatol..

[16] Guo X.-f., Shao X.-f., Li J.-m., Li S., Li K.-l., Li D. (2019). Fruit and vegetable intake and liver cancer risk: A meta-analysis of prospective cohort studies. Food Funct..

[17] Ross A.B., Godin J.P., Minehira K., Kirwan J.P. (2013). Increasing whole grain intake as part of prevention and treatment of nonalcoholic Fatty liver disease. Int. J. Endocrinol..

[18] Kratz M., Marcovina S., Nelson J.E., Yeh M.M., Kowdley K.V., Callahan H.S., Song X., Di C., Utzschneider K.M. (2014). Dairy fat intake is associated with glucose tolerance, hepatic and systemic insulin sensitivity, and liver fat but not β-cell function in humans. Am. J. Clin. Nutr..

[19] Kennedy O.J., Fallowfield J.A., Poole R., Hayes P.C., Parkes J., Roderick P.J. (2021). All coffee types decrease the risk of adverse clinical outcomes in chronic liver disease: A UK Biobank study. BMC Public Health.

[20] Ding Y., Yanagi K., Cheng C., Alaniz R.C., Lee K., Jayaraman A. (2019). Interactions between gut microbiota and non-alcoholic liver disease: The role of microbiota-derived metabolites. Pharmacol. Res..

[21] David L.A., Maurice C.F., Carmody R.N., Gootenberg D.B., Button J.E., Wolfe B.E., Ling A.V., Devlin A.S., Varma Y., Fischbach M.A. (2014). Diet rapidly and reproducibly alters the human gut microbiome. Nature.

[22] Shen F., Zheng R.D., Sun X.Q., Ding W.J., Wang X.Y., Fan J.G. (2017). Gut microbiota dysbiosis in patients with non-alcoholic fatty liver disease. Hepatobiliary Pancreat. Dis. Int..

[23] Sobhonslidsuk A., Chanprasertyothin S., Pongrujikorn T., Kaewduang P., Promson K., Petraksa S., Ongphiphadhanakul B. (2018). The association of gut microbiota with nonalcoholic steatohepatitis in Thais. BioMed. Res. Int..

[24] Weiss J.J., Sanchez L., Hubbard J., Lo J., Grinspoon S.K., Fitch K.V. (2019). Diet quality is low and differs by sex in people with HIV. J. Nutr..

[25] He T., Xu C., Krampe N., Dillon S.M., Sette P., Falwell E., Haret-Richter G.S., Butterfield T., Dunsmore T.L., McFadden W.M. (2019). High-fat diet exacerbates SIV pathogenesis and accelerates disease progression. J. Clin. Investig..

[26] Moshfegh A.J., Rhodes D.G., Baer D.J., Murayi T., Clemens J.C., Rumpler W.V., Paul D.R., Sebastian R.S., Kuczynski K.J., Ingwersen L.A. (2008). The US Department of Agriculture Automated Multiple-Pass Method reduces bias in the collection of energy intakes. Am. J. Clin. Nutr..

[27] Reedy J., Lerman J.L., Krebs-Smith S.M., Kirkpatrick S.I., Pannucci T.E., Wilson M.M., Subar A.F., Kahle L.L., Tooze J.A. (2018). Evaluation of the Healthy Eating Index-2015. J. Acad. Nutr. Diet..

[28] United States Department of Agriculture: Food and Nutrition Service: Center for Nutrition Policy and Promotion (CNPP) How the HEI Is Scored. https://www.fns.usda.gov/how-hei-scored.

[29] Chen Z., Hui P.C., Hui M., Yeoh Y.K., Wong P.Y., Chan M.C.W., Wong M.C.S., Ng S.C., Chan F.K.L., Chan P.K.S. (2019). Impact of preservation method and 16S rRNA hypervariable region on gut microbiota profiling. mSystems.

[30] Drossman D.A. (2006). The functional gastrointestinal disorders and the Rome III process. Gastroenterology.

[31] Sterling R.K., Lissen E., Clumeck N., Sola R., Correa M.C., Montaner J., Sulkowski M.S., Torriani F.J., Dieterich D.T., Thomas D.L. (2006). Development of a simple noninvasive index to predict significant fibrosis in patients with HIV/HCV coinfection. Hepatology.

[32] Bolyen E., Rideout J.R., Dillon M.R., Bokulich N.A., Abnet C.C., Al-Ghalith G.A., Alexander H., Alm E.J., Arumugam M., Asnicar F. (2019). Reproducible, interactive, scalable and extensible microbiome data science using QIIME 2. Nat. Biotechnol..

[33] McMurdie P.J., Holmes S. (2013). Phyloseq: An R package for reproducible interactive analysis and graphics of microbiome census data. PLoS ONE.

[34] Anderson M.J. (2001). A new method for non-parametric multivariate analysis of variance. Austral Ecol..

[35] Dixon P. (2003). VEGAN, a package of R functions for community ecology. J. Veg. Sci..

[36] Armitage E.G., Godzien J., Alonso-Herranz V., López-Gonzálvez Á., Barbas C. (2015). Missing value imputation strategies for metabolomics data. Electrophoresis.

[37] Huber W., von Heydebreck A., Sültmann H., Poustka A., Vingron M. (2002). Variance stabilization applied to microarray data calibration and to the quantification of differential expression. Bioinformatics.

[38] Mock A., Warta R., Dettling S., Brors B., Jäger D., Herold-Mende C. (2018). MetaboDiff: An R package for differential metabolomic analysis. Bioinformatics.

[39] Ruiz-Perez D., Guan H., Madhivanan P., Mathee K., Narasimhan G. (2020). So you think you can PLS-DA?. BMC Bioinform..

[40] United States Department of Agriculture: Food and Nutrition Service: Center for Nutrition Policy and Promotion: National Center for Health Statistics: What We Eat in America/National Health and Nutrition Examination Survey, 2013–2014. Healthy Eating Index-2015, Scores. https://www.fns.usda.gov/healthy-eating-index-hei.

[41] Volpe G.E., Ward H., Mwamburi M., Dinh D., Bhalchandra S., Wanke C., Kane A.V. (2014). Associations of cocaine use and HIV infection with the intestinal microbiota, microbial translocation, and inflammation. J. Stud. Alcohol Drugs.

[42] Tamargo J.A., Sherman K.E., Campa A., Martinez S.S., Li T., Hernandez J., Teeman C., Mandler R.N., Chen J., Ehman R.L. (2021). Food insecurity is associated with magnetic resonance-determined nonalcoholic fatty liver and liver fibrosis in low-income, middle-aged adults with and without HIV. Am. J. Clin. Nutr..

[43] Fukui H. (2019). Role of gut dysbiosis in liver diseases: What have we learned so far?. Diseases.

[44] Weitkunat K., Stuhlmann C., Postel A., Rumberger S., Fankhänel M., Woting A., Petzke K.J., Gohlke S., Schulz T.J., Blaut M. (2017). Short-chain fatty acids and inulin, but not guar gum, prevent diet-induced obesity and insulin resistance through differential mechanisms in mice. Sci. Rep..

[45] Bajaj J.S., Heuman D.M., Hylemon P.B., Sanyal A.J., White M.B., Monteith P., Noble N.A., Unser A.B., Daita K., Fisher A.R. (2014). Altered profile of human gut microbiome is associated with cirrhosis and its complications. J. Hepatol..

[46] Liu Y., Ajami N.J., El-Serag H.B., Hair C., Graham D.Y., White D.L., Chen L., Wang Z., Plew S., Kramer J. (2019). Dietary quality and the colonic mucosa-associated gut microbiome in humans. Am. J. Clin. Nutr..

[47] Yan A.W., Fouts D.E., Brandl J., Stärkel P., Torralba M., Schott E., Tsukamoto H., Nelson K.E., Brenner D.A., Schnabl B. (2011). Enteric dysbiosis associated with a mouse model of alcoholic liver disease. Hepatology.

[48] Palmisano L., Giuliano M., Bucciardini R., Andreotti M., Fragola V., Pirillo M.F., Weimer L.E., Mancini M.G., Vella S. (2009). Modifications of residual viraemia in human immunodeficiency virus-1-infected subjects undergoing repeated highly active antiretroviral therapy interruptions. J. Med. Microbiol..

[49] DallaPiazza M., Amorosa V.K., Localio R., Kostman J.R., Lo Re V. (2010). Prevalence and risk factors for significant liver fibrosis among HIV-monoinfected patients. BMC Infect. Dis..

[50] Human Metabolome Database: Showing Metabocard for Caffeine (HMDB0001847). https://hmdb.ca/metabolites/HMDB0001847.

[51] Tůma P., Samcová E., Balínová P. (2005). Determination of 3-methylhistidine and 1-methylhistidine in untreated urine samples by capillary electrophoresis. J. Chromatogr. B Anal. Technol. Biomed. Life Sci..

[52] Arvind A., Osganian S.A., Cohen D.E., Corey K.E., Feingold K.R., Anawalt B., Boyce A., Chrousos G., de Herder W.W., Dhatariya K., Dungan K., Hershman J.M., Hofland J., Kalra S. (2000). Lipid and Lipoprotein Metabolism in Liver Disease. Endotext.

